# Effects of a work stress intervention on healthcare use and treatment compared to treatment as usual: a randomised controlled trial in Swedish primary healthcare

**DOI:** 10.1186/s12875-020-01210-0

**Published:** 2020-07-06

**Authors:** Christine Sandheimer, Tove Hedenrud, Gunnel Hensing, Kristina Holmgren

**Affiliations:** 1 School of Public Health and Community Medicine, Institute of Medicine, The Sahlgrenska Academy, University of Gothenburg, Gothenburg, Sweden; 2 Department of Health and Rehabilitation, Institute of Neuroscience and Physiology, The Sahlgrenska Academy, University of Gothenburg, Gothenburg, Sweden

**Keywords:** Work-related stress, Primary care, Intervention, Work stress questionnaire (WSQ), Healthcare visits, Healthcare treatment, Healthcare utilisation

## Abstract

**Background:**

Work stress is an increasing burden in society. Identifying early symptoms of work stress in primary healthcare (PHC) could result in earlier and better-targeted care. The Work Stress Questionnaire (WSQ) was developed in PHC for this task. We aimed to evaluate whether the use of the WSQ, in combination with physicians’ feedback, resulted in differences in healthcare visits and treatment compared to treatment as usual (TAU) in patients reporting high stress. Our hypothesis was that patients receiving the intervention would generate more visits to rehabilitation providers during follow-up compared to TAU.

**Methods:**

A two-armed randomised controlled trial was conducted at seven primary healthcare centres (PHCCs) in Region Västra Götaland, Sweden. One group received the WSQ intervention, and the controls received TAU. Employed, not sick-listed persons aged 18–64 years who sought care for mental or physical health complaints at the PHCCs participated. Register data on healthcare visits and treatments 12 months prior to inclusion and 12 months after were obtained and analysed with Fisher’s exact test together with questionnaire data (WSQ and background features).

**Results:**

A total of 271 participants were included in the study, 132 intervention and 139 controls. Visits to psychologists/psychotherapists were higher among intervention participants with high stress (20%, *n* = 87) during follow-up compared to corresponding controls (7%, *n* = 97) (*p* < 0.05). Collaborative care measures were more common among the stressed intervention participants (23%) post-inclusion compared to the stressed controls (11%) (*p* < 0.05). The amount of received cognitive behavioural therapy (CBT) was higher among the stressed intervention group (16%) than among controls (10%) during follow-up.

**Conclusions:**

The intervention group that used the WSQ with physicians’ feedback had an increased number of rehabilitative measures and treatment more in line with established guidelines compared to treatment as usual. Findings of the study indicate that the WSQ can assist in identifying work stress in primary healthcare and contribute to physicians’ recommendations of suitable rehabilitative measures at an earlier stage compared to treatment as usual.

**Trial registration:**

ClinicalTrials.gov. Identifier: NCT02480855. Registered 20 May 2015.

## Background

Common mental disorders (CMDs) such as stress-related ill health, depression and anxiety disorders, are an increasing universal burden not only for the affected individuals but for the society at large [1, 2]. The total costs of mental ill health (CMDs) are estimated at over EUR 600 billion across the 28 European member states [2]. In working age groups, loss of income, labour and productivity, and marginalisation as well as increased strain on the welfare systems, are only a few aspects related to mental ill health and subsequent sickness absence [3–5]. Mental ill health has a multifactorial background, and the workplace or the working situation are in many cases the cause of the ill health [6]. Stress, anxiety or depression is the most mentioned work-related health issue in the majority of the EU countries according to a European survey conducted in 2014 [7].

Previous research has investigated which work factors promote or hinder a good working climate and work health [8–10]. It is well established that high work burden with minimal possibility to control or influence the work situation, as well as existing conflicts, can lead to distress and increases the risk for future ill health [10–12]. Most people with mental distress consult their primary healthcare physician with their symptoms as an initial contact with the health system [13–15]. A Swedish cross-sectional study [16] conducted in primary healthcare (PHC) revealed that 59% of all patients aged 18–65 seeking care at a primary healthcare centre (PHCC), regardless of cause, reported high mental stress levels. Another study found that individuals with continued stress, without possibility for recovery, often seek care repeatedly with an increasing symptom burden before they receive the right care [17]. In some cases, the underlying stress is not identified by the healthcare service until very late in the care process, when sick listing has become unavoidable [17].

There are several questionnaires available that are developed for either screening or diagnostic assessments in the PHC [18]. However, these questionnaires mostly cover depression and/or anxiety and are not aimed towards work-related stress and stressors in the working situation. Since stress that is directly related to the work situation is an increasing issue, there could be benefits, such as earlier and better adjusted care, in introducing a new questionnaire that functions as both a screening tool and a diagnostic instrument in primary healthcare.

The Work Stress Questionnaire (WSQ) was developed in PHC to identify early symptoms of work-related stress in PHC [19]. The WSQ is based on previous research and on the experiences of women on sick leave because of work-related stress [20]. The WSQ takes into consideration organisational and environmental issues at work and individual factors such as high commitment and engagement in work. Earlier studies with the WSQ conducted in PHC showed that the questionnaire predicted future sick leave at follow-up among the participants who scored high stress levels at baseline [21]. The next step was therefore to evaluate the practicability of the WSQ as a screening and diagnostic instrument for physicians in their consultation and treatment of their patients [22].

In Sweden, the healthcare system is tax-funded with universal coverage for the whole population. Private care providers are also mainly tax-funded and thus reimbursed by the county. There are two levels of care: primary healthcare (including child, youth and maternity healthcare) and specialist hospital care (outpatient and inpatient care). Each patient needs to pay a fee (out-of-pocket payment), currently EUR 9.5 (SEK 100) for a visit to a PHC physician or psychotherapist, EUR 7.6 (SEK 80) for a visit to an occupational therapist or physiotherapist and EUR 28.4 (SEK 300) for visits to specialist outpatient care. Patient fees are the same in both public and private PHCCs. There is protection against high costs for outpatient care; when the patient has accumulated out-of-pocket payments of EUR 108.9 (SEK 1150) during a 12-month period, further healthcare visits during that time period are free of charge for the individual.

Our aim with this study was to evaluate whether responding to the WSQ in combination with feedback of the results from the physician would lead to differences in healthcare use and treatment compared to treatment as usual (TAU) in patients reporting high stress. Our hypothesis was that patients receiving the intervention (responding to the WSQ plus physician’s feedback) would generate more visits to rehabilitation providers, that is, psychologists/psychotherapists and occupational therapists/physiotherapists, during the year after inclusion compared to control patients receiving TAU. Our aim was also to assess whether the intervention would result in care measures more in line with established national treatment guidelines.

## Methods

### Study context

This study is part of the research project ‘Early Identification of Work-related Stress’ titled TIDAS in the research programme “New ways: Mental health at work” at the Sahlgrenska Academy, University of Gothenburg (www.epso.gu.se/newways).

### Study setting

This study was conducted in the primary healthcare in South-West Sweden in the Region Västra Götaland (VGR). VGR, with its 1.7 million residents (approx. 17% of the Swedish population), has about 200 PHCCs, of which 52% are public and 48% are private (both are reimbursed by VGR). During 2017, 71% of the population in VGR visited the PHC at least once [23]. On average, 3.6 visits per inhabitant were made to the PHC, which corresponds to over 4 million visits to the region’s PHCCs [23].

### Study design and recruitment

This study was designed as a randomised controlled trial (RCT) in accordance with the CONSORT® guidelines [24]. The RCT study was conducted at public and private PHCCs in VGR and is presented in full in a previously published study protocol [22]. Recruitment of the PHCCs was conducted between May 2015 and November 2015. Fifty-one of 200 PHCCs were asked to participate in the study. Seven PHCCs accepted participation (four public and three private). The main reasons for the high number of refusals from the PHCCs were either that the PHCC had implemented primary care triage (in which a nurse assesses each patient case and triages the patient to a suitable care provider), or had a lack of time, and/or already had an ongoing research project. A research assistant on site was responsible for identification and recruitment of participants. The PHCCs were financially reimbursed for each patient, both intervention and control, recruited to the study.

### Study population

As research has found that work-related stressors result in numerous mental and physical health complaints [25, 26], the target group for this study were non-sick-listed employed persons between 18 and 64 years of age who sought care for physical and/or mental complaints at their PHCC [27–29]. In line with the purpose of the RCT to early identify people with work-related stress, patients with established diagnoses were excluded. The full inclusion and exclusion criteria are listed in Table 1.

**Table 1:** Inclusion and Exclusion Criteria

**Inclusion criteria**	
Patients seeking care for:	
• depression	
• anxiety	
• musculoskeletal disorders	
• gastro-intestinal	
• cardiovascular conditions	
• other potentially stress related symptoms	
**Exclusion criteria**	
Patients with:	
• ≥7 days sickness absence the last month	
• sickness or activity benefits	
• ongoing pregnancy (due to risk of pregnancy-related healthcare contacts)	
Patients seeking care for:	
• psychiatric conditions (e.g. schizophrenia, bipolar disorder)	
• diabetes	
• urinary tract infection (UTI)	
• infections (cold, sore throat)	
• fractures	
• lumps and spots	
• allergy	
• prolonging of sick leave	
• medical check-ups	
• chronic obstructive pulmonary disease (COPD)	

### The work stress questionnaire (WSQ)

The instrument in focus for this study was the WSQ. The WSQ was developed from a qualitative study of former long-term sick-listed women who emphasised the combined importance of organisational and individual factors for the progression from health to sick leave [20]. The WSQ has been used in PHC studies [21] and population studies [30]. The psychometric properties of the WSQ have been assessed with test-retest reliability and face validity with satisfying results [19, 31].

The questionnaire consists of 21 questions grouped into four categories. The categories aim to identify stress in relation to (1) Influence over work situation, (2) Indistinct organisation and conflicts, (3) High work commitment, and (4) Work interference with leisure time. Responses to each item are given on a four-point ordinal scale: “Not at all stressful”, “Less stressful”, “Stressful”, and “Very stressful”. For more details, see Holmgren et al. 2009 [19] and 2016 [22].

### Procedure

#### The randomisation

Randomisation was done on the physician level meaning that the physicians at the same participating PHCCs were randomised to either conduct the intervention or belong to the control group. After the randomisation of the physicians, the research team visited the PHCCs and presented the study to the staff and informed them which physicians were randomised to each group.

#### Pre-intervention training

The intervention physicians got a short training about the WSQ, on how to interpret the WSQ and how to give feedback based on the WSQ’s result. Moreover, the intervention physicians received a list of healthcare providers and other relevant rehabilitation providers for possible patient referrals. The research team stressed that the intervention physician first should address the health complaint for which the patient sought care and as a next step address the result from the WSQ, and, if work-related stress was identified, give recommendations for further care (like referrals to other healthcare professions). The short training was given either one on one or in a group depending on what the physicians’ schedule permitted. The physicians randomised to the control group were instructed to carry on as usual, that is, giving the care that the patients were entitled to.

#### The intervention

The research assistant on site was given permission from the head of the PHCC to, in collaboration with personnel, identify eligible patients from the electronic patient record system. After identifying possible eligible patients (based on the inclusion and exclusion criteria), the research assistant or the personnel contacted the patients, either on site or by telephone, and gave information about the study and invited them to participate. If the patients were interested in participating in the study, they gave their informed written consent. Patients who had an appointment with an intervention physician filled in the WSQ questionnaire before the consultation, together with some questions on background characteristics. The WSQ was compiled by the research assistant and given to the treating physician. If work-related stress was identified, the intervention physician gave recommendations for further care, departing from the patient’s illness and from the specific work-related problems detected by the WSQ. If the results from the WSQ indicated lack of stress in all four categories, no further feedback from the intervention physician was given.

Participants who had an appointment with a control physician received TAU and then filled in the questionnaire (WSQ and background characteristics) after their visit to the physician. This was done to make sure that we later could compare the controls with the intervention participants.

### Intervention period

The intervention periods were 4 to 8 weeks at each PHCC and lasted from May 2015 to the end of January 2016. An exception was one PHCC that had two intervention periods over a period of 12 weeks.

### Data

Data from the questionnaire, filled in by the patients at the PHCC, included both the WSQ and information on background characteristics such as age, sex, employment status et cetera. Data on healthcare contacts were obtained from VGR’s healthcare database, VEGA, which includes information on hospital and primary care consumption by VGR inhabitants both within and outside the region. The objective of the database is to be a tool for uniform care assessment and follow-up. The information collected consisted of healthcare level, private or public management, inpatient care, care provider, number of visits and care measures. Healthcare levels included PHC, specialised healthcare at a county hospital and national/regional specialised healthcare. Due to having few participants in the category national/regional specialised healthcare, this healthcare level was excluded from the analyses but is available from the authors on request. We excluded measures not relevant for our research question and those that were listed as exclusion criteria at inclusion (Table 1). Patients seeking care for these measures were not the target group for this study. The categorisation of care measures was based on the guidelines from the National Board of Health and Welfare (Socialstyrelsen) and was divided into four categories: (1) Diagnostics/Assessments, (2) Treatment, (3) Information/Education and (4) Collaborative Care.

One participant in the intervention group had an incorrect/not interpretable social security number and therefore had only self-reported baseline data and no register data. Data from 12 months pre-inclusion and from index date and 12 months post-inclusion were collected. The register data was merged with the questionnaire data.

### Statistical analysis

This study applied methods commonly used for descriptive statistics. For the analyses, Fisher’s exact test was performed for a group-wise comparison. Subgroup analyses were made with the participants (in both groups) reporting high stress in order to distinguish the effect of the intervention. Because of the very skewed data material, the presentation of the data is in proportions instead of means or medians. The software used for the statistical analyses was IBM SPSS Statistics® version 25. The level of statistical significance was set at *p* < 0.05 (95%).

### Ethics

Prior to the inclusion, each patient was given oral and written information from the research assistant about the study and the possibility of withdrawing from participation at any stage without jeopardising their care at the PHCC. Each participating patient gave informed consent. This study was approved by the regional ethical review board in Gothenburg, Sweden (Dnr: 125–15; T131–17). Registered at ClinicalTrials.gov (Identifier: NCT02480855).

## Results

**Fig. 1:**
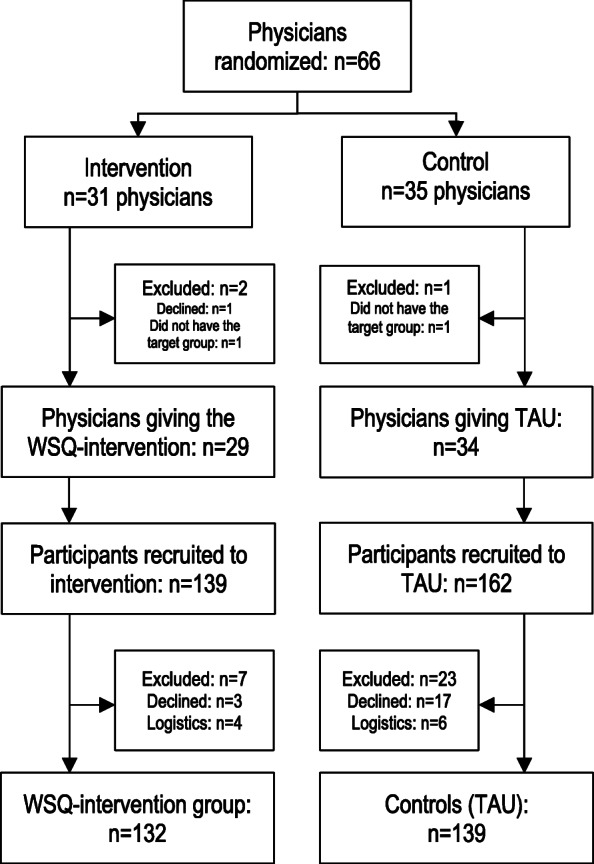
Flowchart of recruited physicians and participants

Information about number of involved physicians and participating patients is found in Fig. 1. Descriptive characteristics of the study participants is presented in Table 2. Hereafter the participants who received the intervention will be referred to as WSQ-IG (WSQ intervention group), and the participants who received TAU will be referred to as controls.

**Table 2:** Baseline characteristics of study participants^a^, data from self-reported questionnaire, *n* = 271

	WSQ-IG^b^ % (*n* = 132)	Controls % (*n* = 139)
Sex		
Women	67 (88)	70 (97)
Men	33 (44)	30 (42)
Age categories		
19–30 years	16 (21)	19 (26)
31–50 years	44 (58)	54 (76)
51–64 years	40 (53)	27 (37)
Civil status		
Single	25 (33)	18 (25)
Married/cohabitant	69 (91)	76 (106)
In a relationship, live-apart	5 (7)	5 (7)
Educational level		
Compulsory schooling	10 (13)	11 (15)
Secondary school	46 (61)	42 (59)
University or higher	44 (57)	47 (65)
Occupational class		
Skilled/unskilled manual	37 (49)	42 (58)
Medium/low non-manual	46 (60)	41 (56)
High-level non-manual	17 (23)	17 (24)
Main type of employment		
Employed until further notice	79 (105)	80 (112)
Project employee	2 (3)	1 (1)
Substitute	2 (3)	3 (4)
Employed by the hour	4 (5)	5 (7)
Self-employed	6 (8)	5 (7)
Other type of employment	6 (8)	5 (7)
Scope of work		
Full-time	76 (100)	77 (107)
Part-time, ≥ 15 h/week	24 (31)	22 (30)
Reason for consultation^c^		
Mental or behavioral	57 (75)	50 (69)
Musculoskeletal	47 (62)	32 (44)
Gastrointestinal	20 (26)	20 (28)
Cardiovascular	12 (16)	11 (16)
Other	22 (29)	19 (27)
High WSQ-values^d^		
Low influence over work situation	41 (54)	39 (54)
Indistinct organisation and conflicts	21 (28)	19 (26)
High work commitment	48 (63)	45 (62)
Work interference with leisure time	41 (54)	40 (55)

^a^Some baseline characteristics are also published in the study protocol by Holmgren et al. [22]

^b^WSQ-IG = the group that received the WSQ intervention

^c^Multiple answers were possible

^d^Participants that scored values of 3 (stressful) and 4 (very stressful) on the Work Stress Questionnaire (WSQ)

### Perceived stress among the participants

No distinct differences in perceived stress between the WSQ-IG and controls were found in either of the two time periods (Table 2). The highest frequency of perceived stress, among both groups, was in the category *High work commitment* (63% among WSQ-IG and 62% among controls).

### Healthcare use among participants with perceived stress

No significant differences in types of healthcare use (i.e. healthcare level, public/private care, outpatient/inpatient care) were found between the WSQ-IG participants and the controls that reported high perceived stress.

Information on visits to different care providers among the participants with perceived stress can be found in Table 3. Visits to psychologist/psychotherapist was significantly higher among the stressed WSQ-IG (20%) in the post-inclusion period compared to the stressed controls (7%) (*p* < 0.05). Visits to occupational therapists and physiotherapists were 43% among the WSQ-IG with high stress levels and 32% among the corresponding controls during follow-up. The difference between the two groups was not statistically significant (*p* = 0.38).

**Table 3:** Visits to care providers among WSQ intervention group and controls reporting high stress^a^

	Before inclusion (< 12 months)			Follow-up (≥12 months)		
	WSQ-IG % (*n* = 84^b^)	Control % (*n* = 92^b^)	*p*-value	WSQ-IG % (*n* = 87^b^)	Control % (*n* = 97^b^)	*p*-value
Visits to						
Physician			.94			.38
0	20 (17)	22 (20)		3 (3)	0 (0)	
1–5	55 (46)	50 (46)		48 (42)	47 (46)	
6–10	16 (13)	17 (16)		25 (22)	27 (26)	
> 11	10 (8)	11 (10)		23 (20)	26 (25)	
Nurse			.32			.55
0	6 (5)	3 (3)		13 (11)	13 (13)	
1–5	71 (60)	67 (62)		55 (48)	54 (52)	
6–10	14 (12)	24 (22)		17 (15)	24 (23)	
> 11	8 (7)	5 (5)		15 (13)	9 (9)	
Psychologist/Psychotherapist			.86			.048^†^
0	94 (79)	96 (88)		81 (70)	93 (90)	
1–5	5 (4)	4 (4)		12 (10)	6 (6)	
6–10	1 (1)	0 (0)		2 (2)	0 (0)	
> 11	0 (0)	0 (0)		6 (5)	1 (1)	
Occupational therapist/Physiotherapist			.59			.38
0	63 (83)	73 (102)		58 (50)	68 (66)	
1–5	17 (23)	11 (15)		15 (13)	8 (8)	
6–10	4 (5)	6 (8)		7 (6)	7 (7)	
> 11	9 (12)	5 (7)		21 (18)	17 (16)	

^a^Participants that scored values of 3 (stressful) and 4 (very stressful) in at least one of the four categories in the WSQ

^b^Number of valid observations

Fisher’s Exact Test was performed with a significance level of .05 (95%).

^†^Statistically significant difference (*p* < 0.05) between groups

### Healthcare treatment among participants with perceived stress

Treatment was the most frequent care measure category in both groups with high stress levels irrespective of time period (12 months pre-inclusion and from index date and 12 months after) (Table 4). The WSQ-IG with perceived stress received much more collaborative care measures during follow-up (23% vs 2% before inclusion). Among the stressed controls, this category changed from 2 to 11%. The inter-comparison analysis resulted in a statistically significant difference between the two groups (*p* < 0.05).

**Table 4:** Healthcare treatments among WSQ intervention group and controls reporting high stress^a^

	Before inclusion (< 12 months)			Follow-up (≥12 months)		
	WSQ-IG % (*n* = 84^b^)	Control % (*n* = 92^b^)	*p*-value	WSQ-IG % (*n* = 87^b^)	Control % (*n* = 97^b^)	*p*-value
Healthcare treatment						
Diagnostics/Assessments	8 (7)	8 (8)	1	17 (15)	12 (12)	.41
Treatment	10 (8)	14 (13)	.36	28 (24)	26 (25)	.87
Information/Education	4 (3)	4 (4)	1	9 (8)	5 (5)	.39
Collaborative care	2 (2)	2 (2)	1	23 (20)	11 (11)	.048^†^

^a^Participants that scored values of 3 (stressful) and 4 (very stressful) in at least one of the four categories in the WSQ

^b^Number of valid observations

Fisher’s Exact Test was performed with a significance level of .05 (95%).

^†^Statistically significant difference (*p* < 0.05) between groups

*Information and counselling with patient over telephone* was the most common care measure among the WSQ-IG and controls with high perceived stress in the pre-inclusion period (26% among WSQ-IG and 12% among controls) (Additional file 1). In the WSQ-IG, the most frequent care measure during follow-up was *Cognitive behavioural therapy* (CBT) (16% compared to 5% before inclusion). For the controls, amount of CBT had not changed between the two time periods and was about 10%.

## Discussion

In this study we investigated whether the patients with perceived high stress, as measured by the WSQ, and receiving feedback from a physician would have more rehabilitative healthcare visits and treatments post-intervention compared to controls receiving TAU. Significant differences were found between the WSQ intervention group and controls who reported high perceived stress, in visits to psychologists and in amount of received collaborative care. A difference between the two groups was also found in amount of received CBT. This confirms our hypothesis that the intervention with physicians giving feedback from the WSQ can result in increased numbers of rehabilitative measures at an earlier stage in the care process compared to TAU. However, with a small sample size, caution must be applied, as the findings might not be robust or persist in future studies with larger study populations or in other healthcare systems.

### Findings in relation to established national treatment guidelines

According to both the Swedish and British national guidelines, evidence-based treatments for CMDs are psychological measures such as CBT and interpersonal therapy, among others [32, 33]. Furthermore, in the latest and revised national guidelines for treatment of CMDs [32] the Swedish National Board of Health and Welfare emphasised *collaborative and structural care by a care manager* as a point of recommendation for treatment of patients diagnosed with a CMD.

In other words, we could see that the intervention group reporting high perceived stress levels did receive care measures more in line with established clinical treatment guidelines compared to the stressed controls. This indicates that adherence to guidelines could be more effective when a CMD has been identified by the physician, as also confirmed in a study by Smolders et al. [34].

### Comparison with existing literature

To the best of our knowledge, there have been no or few studies that have investigated the use of a self-administered work stress questionnaire in combination with physicians’ feedback in relation to our outcomes: healthcare use and treatment.

Our first major finding was the higher frequency of visits to psychologists/psychotherapists among the stressed WSQ-IG. This result indicates that the intervention could have improved the physicians’ recognition of patients in need of psychological treatment. Our findings reflect those of Magruber-Habib et al. [35] who showed that the recognition rate of patients with depression increased when physicians used a screening instrument (for depression) with feedback of the results, with more initiated treatments as an effect.

Another finding relating to the previous one, was the higher amount of received CBT among the stressed WSQ-IG compared to the stressed controls. As the WSQ-IG participants had more visits to psychologists/psychotherapists, it is logical that this group also received more CBT treatment, as psychologists/psychotherapists are the ones authorised to give the treatment.

Our study also showed that the WSQ-IG with high stress had a higher proportion of received collaborative care compared to the corresponding controls. Collaborative care has in an earlier review study [36] been shown to be an important part for a successful recognition and management of depression in studies that used screening questionnaires in combination with feedback of results.

Finally, one finding that was not statistically significant was the difference in frequency of visits to occupational therapists and physiotherapists between the two groups. The visits to occupational therapists/physiotherapists increased in both groups reporting high stress levels, during the 12-month follow-up period. The increase appeared to be greater among the WSQ-IG, although no statistical significance was reached. In 2014, Sweden introduced a new healthcare reform called “Vårdval Rehab” (Choice of care rehab) [23]. This reform aimed to increase the access to rehabilitative care providers, as the population could then freely choose a rehabilitation provider without the need of a referral note from their physician. Since then, there has been a gradual increase each year in visits to rehabilitation providers such as occupational therapists and physiotherapists [23]. The introduction of this reform could partly explain the overall increase in visits among both participant groups, but not the seemingly higher increase among the intervention group.

The observed differences between the intervention group and the controls could be explained by the fact that the intervention led to the physicians being more observant/aware of the patients’ problems and need of care. This could, in turn, have resulted in the physicians referring the patients to more suitable treatments of which they (the physicians) have had good experience and which are available in the PHC organisation.

An earlier study has shown that through a structured approach, physicians become aware of their patients’ occupations and of factors in the working situation that can cause stress, and that by becoming aware of work-related risks of ill health, physicians can advise their patients and refer them to more adjusted measures and to other care providers [37].

### Strengths and limitations

A strength of the study is the predefined study protocol outlined in accordance with strictly controlled guidelines, in this case the CONSORT guidelines [24]. Another strength is the use of register data for follow-up. The strength of using data on healthcare contacts from the VEGA database is that it is systematic, comprehensive and up to date (data are updated on a monthly basis). The PHCCs with contracts with the region (VGR) have an obligation to send in their patient information for the database. The database is one of a kind in Sweden when it comes to collecting PHC data.

A possible limitation with our choice to randomise on the physician level could be the spillover effect, meaning that intervention physicians could talk about the study procedure with their colleagues randomised to the control group. We anticipated this risk to be low, as the physicians are autonomous and work independently from one another, and because of the brief intervention that was imbedded in their everyday routine. Also, the time period at each centre was short. The reason behind this randomisation was to avoid large variations in sociodemographic and socioeconomic factors between participants. Including both intervention and control physicians at the same PHCC would minimise this risk and was therefore seen as the more advantageous choice.

Another methodological consideration important to acknowledge is the lack of data from the occupational health service. Since our target group were employed persons with possible work-related stress, we can conclude that some of the participants could have been referred to their occupational health service (OHS) by the primary care physician. The VEGA database does not cover healthcare data from occupational providers, and there are no other databases in Sweden that collect data from these care providers. The lack of follow-up data from the OHS could have influenced our result, as measures relevant for our study could have been prescribed to patients in the OHS and not in ordinary healthcare. As a result, the effect of the intervention risks being attenuated. However, only about 65% of the working population have access to an OHS, with one third of these only having access “on paper” [38]. Therefore, we believe the risk of a weaker intervention effect to be rather small.

All in all, we found only small differences between the WSQ intervention group who perceived themselves as stressed and the control group (with stress) who received TAU. The lack of statistically significant differences in most of the analyses between the intervention group and the controls could be because the compared groups were quite small, which affects the likelihood of identifying existing significant differences. As a further consequence of the small sample, there were limitations in the available choices of statistical methods and also in the possibility to conduct stratified analyses on different background characteristics.

Although we saw an effect regarding differences in healthcare treatment between the intervention group and the controls, we cannot say with absolute certainty what actually caused the effect. In another published article [39] based on the same RCT as this one, with the outcome measure *number of sick leave days*, no differences were detected. Possible explanations for the lack of effect in that study were low power or that the time period for follow-up was too short [29]. Thus, there is a need for future studies to investigate this effect with larger study samples, which also would enable more in-depth analyses.

## Conclusions

The intervention group that used the WSQ with physicians’ feedback had an increased number of rehabilitative measures and treatment more in line with established guidelines compared to treatment as usual. Findings of the study indicate that the WSQ can assist in identifying work stress in primary healthcare and contribute to physicians’ recommendation of suitable rehabilitative measures at an earlier stage compared to treatment as usual.

## Supplementary information

**Additional file 1: Table S1.** Most frequent care measures^*^ in outpatient care among WSQ intervention group and controls reporting high stress^**^.

## Data Availability

The datasets generated and/or analysed during the current study are not publicly available due to Swedish law but are available from the corresponding author on reasonable request.

## Abbreviations

CBT: Cognitive behavioural therapy
CMD: Common mental disorder
OHS: Occupational health service
PHC: Primary healthcare
PHCC: Primary healthcare centre
RCT: Randomised controlled trial
TAU: Treatment as usual
VGR: Region Västra Götaland
WSQ: Work Stress Questionnaire
WSQ-IG: WSQ intervention group
