# Supplementary material for: Effects of a work stress intervention on healthcare use and treatment compared to treatment as usual: a randomised controlled trial in Swedish primary healthcare
Source: BMC Fam Pract. 2020 Jul 6;21:133. doi: 10.1186/s12875-020-01210-0 (PMC7339485; doi:10.1186/s12875-020-01210-0)
Supplement: Supplementary file 1 — Additional file 1: Table S1. Most frequent care measures* in outpatient care among WSQ intervention group and controls reporting high stress**. [file 12875_2020_1210_MOESM1_ESM.docx]

| Table 4. Most frequent care measures^*^ in outpatient care among WSQ intervention group and controls reporting high stress^**^ | | | | | | |
| --- | --- | --- | --- | --- | --- | --- |
|  | WSQ-IG  Before inclusion (<12 months), n=84^a^ | | | Control  Before inclusion (<12 months), n=92^a^ | | |
| Top ten  care measures | Code | Description | % (freq) | Code | Description | % (freq) |
| 1 | XS012 | Information, counseling (phone) | 26 (71) | XS012 | Information, counseling (phone) | 12 (100) |
| 2 | QG003 | Muscle function and weight training | 6 (17) | DU011 | Cognitive-Behavioural treatment | 10 (89) |
| 3 | DU011 | Cognitive-Behavioural treatment | 5 (14) | DA001 | Acupuncture | 9 (86) |
| 4 | QV011 | Training programme | 4 (11) | UV005 | Medical training therapy | 6 (57) |
| 5 | QJ000 | Support and training in learning and appliance of knowledge | 4 (10) | QV011 | Training programme | 5 (43) |
| 6 | GB002 | Information and education | 3 (9) | UV110 | Multimodal rehabilitation level 1 | 5 (43) |
| 7 | UV012 | Assessment of need of physiotherapy | 3 (9) | XV001 | Pool training | 4 (38) |
| 8 | XV007 | Follow-up, control of treatment | 3 (9) | UV117 | Coordination of rehabilitation | 4 (37) |
| 9 | QK000 | Support and training in handling demands | 3 (8) | XV007 | Follow-up, control of treatment | 4 (35) |
| 10 | DU007 | Supporting conversation | 2 (5) | QK005 | Stress management | 3 (27) |
|  |  |  |  |  |  |  |
|  | Follow-up (≥12 months), n=87^a^ | | | Follow-up (≥12 months), n=97^a^ | | |
| 1 | DU011 | Cognitive-Behavioural treatment | 16 (57) | XS012 | Information, counseling (phone) | 10 (96) |
| 2 | XS012 | Information, counseling (phone) | 13 (48) | DU011 | Cognitive-Behavioural treatment | 10 (92) |
| 3 | XV007 | Follow-up, control of treatment | 6 (22) | UV005 | Medical training therapy | 6 (59) |
| 4 | DU007 | Supporting conversation | 5 (20) | QV011 | Training programme | 5 (47) |
| 5 | DA001 | Acupuncture | 5 (18) | DA001 | Acupuncture | 4 (42) |
| 6 | QG003 | Muscle function and weight training | 4 (15) | QG003 | Muscle function and weight training | 4 (40) |
| 7 | UV012 | Assessment of need of physiotherapy | 4 (13) | DU007 | Supporting conversation | 4 (39) |
| 8 | QV011 | Training programme | 3 (12) | QG001 | Mobility training | 4 (38) |
| 9 | UV005 | Medical training therapy | 3 (12) | AU011 | Estimation of mental symptoms | 4 (34) |
| 10 | UV110 | Multimodal rehabilitation level 1 | 3 (12) | UV012 | Assessment of need of physiotherapy | 3 (26) |
| ^*^Only the ten most frequent care measure codes are listed, the rest of the included care measures and their frequencies are available from the authors on request  ^**^Participants that scored values of 3 (stressful) and 4 (very stressful) in at least one of the four categories in the WSQ  ^a^Number of valid observations | | | | | | |
